# Connecting minds and catalyzing collaboration: the interest groups of health technology assessment international

**DOI:** 10.1017/S0266462325100287

**Published:** 2025-07-07

**Authors:** Antonio Migliore, Nicola Vicari, George Valiotis, Ann Single

**Affiliations:** Health Technology Assessment International (HTAi), Edmonton, AB, Canada

**Keywords:** health technology assessment, collaboration, interest groups, multidisciplinary, stakeholder involvement

## Abstract

Health Technology Assessment international (HTAi) supports global collaboration and innovation in HTA through its dynamic network of Interest Groups (IGs). These thematic communities provide a dedicated platform for members to engage in focused, collaborative efforts that drive professional exchange, advance methodologies, and develop best practices in HTA. This commentary offers a panoramic overview of all IGs, their evolution, aim, and initiatives. By drawing on diverse stakeholder perspectives, spanning academia, clinical practice, industry, and patient communities, the IGs foster inclusiveness and extend HTAi’s influence to significantly contribute to the broader HTA community. Through activities such as workshops, conference sessions, webinars, publications, and research projects, they offer opportunities for professional development and thought leadership. The IGs’ cross-cutting contributions position them as engines of innovation to ensure HTAi remains at the forefront of shaping a globally relevant, responsive, and ethically grounded HTA ecosystem.

## Background

Health Technology Assessment (HTA) is a multidisciplinary process that uses explicit methods to determine the value of a health technology at different points in its lifecycle with the purpose of informing decision making to promote an equitable, efficient, and high-quality health system. Released in 2020, this definition was derived from a collaboration among the major associations in the field (1). Understanding the actual magnitude of HTA implies disclosing some of the keywords included in its definition: health technology, explicit methods, value, and technology lifecycle. Health technologies encompass a broad range of interventions, including medicines, medical devices, diagnostic tests, surgical procedures, rehabilitation programs, and healthcare delivery models. To provide critical insights to inform decision making, HTA needs to use solid methodological frameworks, which need to be systematic, transparent, and evidence-based. A key to HTA’s success lies in its ability to examine health technologies from multiple dimensions of value. The evaluation extends beyond clinical outcomes to include not only health economic impact but also patient preferences, ethical and social considerations, legal and regulatory implications, organizational impact, and environmental sustainability. Additionally, HTA integrates perspectives from diverse stakeholders, acknowledging that the perceived value of a technology may differ depending on who is affected and the specific decision-making context, and can be applied across the technology lifecycle.

Health Technology Assessment international (HTAi) is the global society for the promotion of HTA. HTAi was officially established in 2003 in Canada, following the closure of the International Society for Technology Assessment in Health Care, which was formed in 1985 but liquidated in 2003 due to financial issues (2).

HTAi Interest Groups (IGs), formerly known as Interest Subgroups (ISGs), are specialized communities within the society focused on key HTA-related topics. They provide a dedicated platform for members to engage in focused, collaborative efforts that drive professional exchange, advance methodologies, and develop best practices in HTA. Recognizing the diverse expertise of its global membership, HTAi established these groups to share knowledge, develop resources, conduct research, and influence policy discussions. Since the inception of the first IG in 2005, the IGs have fostered interdisciplinary dialogue by allowing each group to concentrate on a specific thematic area aligned with members’ shared professional interests. Today, IGs’ size ranges from about 100 to more than 650 members, and about 70 percent of HTAi members belong to one or more IGs. By engaging policy makers, researchers, industry stakeholders, and healthcare practitioners, the IGs extend HTAi’s influence beyond individual members and significantly contribute to the broader HTA community. Through activities such as workshops, publications, and conference sessions, they offer opportunities for professional development and thought leadership, ensuring that the society remains at the forefront of global HTA advancements. Empowering members to shape the field, the IGs encourage collaboration to address pressing healthcare challenges, leveraging a unique multistakeholder membership for collaborative action. These groups continue to evolve, meeting the dynamic needs of the HTA landscape and reinforcing HTAi’s worldwide mission.

## Interest groups

The following sections introduce the eleven HTAi IGs, presenting their respective aims and relevance to HTA. Table 1 presents a summary of their aim and the number of members and represented counties.

**Table 1.** The eleven Interest Groups within Health Technology Assessment international (HTAi)

Name	Acronym	Year established	Aim(s)	Number of members^a^	Countries represented^a^
Disinvestment and early awareness	DEA IG	2014	(i) To promote collaboration and sharing of knowledge and expertise on horizon scanning, early awareness, and disinvestment across the technology lifecycle; (ii) To develop methods for prioritizing and assessing emerging, obsolete, or low-value technologies; (iii) To promote practical application of de-adoption or disinvestment by health systems.	209	43
Early-career network	ECN	2011	(i) To connect and facilitate networking among members who are early in their HTA careers; (ii) To promote standardized curricula, develop mentorship programs, and advocates formal training pathways in HTA.	172	45
Ethical issues in HTA	EIG	2005	(i) To establish and strengthen connections with others in related fields, e.g., patient and citizen involvement in HTA and the role of philosophy in HTA; (ii) To share good practices and related expertise in methodologies and tools for identifying and addressing ethical issues in HTA.	185	43
Hospital-based HTA	HB-HTA IG	2006	(i) To provide a dialogue opportunity for members who are producers or users of HTA within hospitals; (ii) To promote awareness and knowledge of hospital-based HTA by sharing its principles, values, methods, and best practices; (iii) To identify trends to support integration of national and hospital-based HTA programs.	304	53
HTA in developing countries	DCIG	2008	(i) To share members’ experiences, activities, resources, and opportunities for education and training; (ii) To discuss ideas and projects, needs, challenges and solutions that are specific to the developing countries.	392	68
Information retrieval	IRG	2005	(i) To advance literature searching and synthesis by exchanging information, sharing expertise, processes, methodology, and tools.	111	40
Medical devices	MDIG	2019	(i) To promote collaboration, sharing knowledge, expertise, and development of methods for prioritizing and assessing medical devices; (ii) To discuss the strengths, weaknesses, challenges, and opportunities of introducing medical device innovation into healthcare systems under the responsibility of increasing patient outcomes and lowering system costs.	400	40
Patient and citizen involvement in HTA	PCIG	2005	(i) To promote and develop robust methodologies to incorporate patient and citizen perspectives into HTA and share best practices in patient and citizen participation in the HTA process; (ii) To strengthen HTA by systematic incorporation of patient and citizen perspectives; (iii) To support countries with limited experience of HTA to elicit and incorporate patient and citizen perspectives.	341	49
Public health	PHIG	2019	(i) To raise awareness on the importance of HTA to evaluate public health interventions for promoting health, preventing diseases, and preserving and/or ensuring sustainable healthcare and social infrastructures; (ii) To facilitate the development and application of appropriate methodologies and HTA approaches in the area of public health; (iii) To engage with diverse stakeholders to strengthen intrasectorial cooperation of major importance in the area of HTA and public health.	430	62
Rare diseases	RDIG	2023	(i) To share good practices in the generation and interpretation of evidence in rare disease populations; (ii) To explore how HTA can consider wider aspects of value in rare disease populations within the context of fairness to society.	337	50
Real-world evidence and artificial intelligence	RWE&AI IG	2020	(i) To provide a network for education, communication, collaboration, and thought leadership among its members on strategies and methodologies that allow supporting HTA through the use of real-world evidence, including the use of artificial intelligence.	669	64

a Membership data at December 2024.

### Disinvestment and early awareness

The Disinvestment and Early Awareness IG (DEA IG) is dedicated to: (i) collaboration, sharing knowledge, and expertise; (ii) development of methods for prioritizing and assessing emerging, obsolete, or low-value technologies; and (iii) the practical application of de-adoption or disinvestment by health systems. It was established in 2014 under a memorandum of understanding between HTAi and the EuroScan International Network, a pioneer in the field and long-standing partner of HTAi. The DEA IG focuses on horizon scanning, early awareness, and disinvestment across the technology lifecycle (3;4). The lifecycle of technologies spans initial development, market introduction, widespread adoption, potential obsolescence, and eventual withdrawal (5). Early awareness is essential for identifying and assessing emerging technologies before they become widely adopted. By proactively identifying promising technologies, healthcare systems can inform decisions about their adoption, prioritizing those with demonstrated clinical and economic benefits. Early awareness and alert systems are designed to identify innovative technologies with the potential for significant impacts on healthcare. At the other end of the spectrum, disinvestment is intended to ensure that technologies that are outdated, ineffective, or no longer cost-effective are removed from practice. European HTA agencies have increasingly recognized the importance of disinvestment as a strategy to improve healthcare efficiency (6). In Italy, for example, structured frameworks have led to more efficient spending (7). Others have proposed evidence-based frameworks for identifying technologies of no- or low-added value (8). Nevertheless, disinvestment is often impeded in practice by the inertia of long-standing or routinized administrative, clinical, and payment practices, as well as various ethical, political, and economic concerns.

### Early career network

The HTAi Early Career Network (ECN IG) was established in 2011 to connect and facilitate networking among three main groups: members who are early in their HTA careers, including recent graduates of programs in HTA or such related fields as epidemiology, health economics, and public health; professionals who are approaching HTA later in their careers; and experienced HTA professionals. Among its functions, the ECN IG promotes standardized curricula, develops mentorship programs, and advocates formal training pathways in HTA. The “Newcomer’s Guide to HTA,” a recent initiative of this IG, comprises a curated collection of resources for navigating the HTA landscape, ranging from introductory overviews to detailed methods and processes (9). Another ongoing initiative of the ECN IG is a mentoring program that matches senior members of the society with newcomers.

As HTA continues to evolve in response to technological advancements, policy shifts, and healthcare challenges, there is a growing need to ensure that early career professionals receive the necessary training, mentorship, and resources to develop their expertise. One of the key challenges in HTA education is the lack of standardized competencies and training programs across different regions and institutions. This includes a common understanding of HTA competencies to enhance educational and training programs globally, supported by a well-defined competency framework to help early career professionals navigate the multidisciplinary nature of HTA (10). In addition to technical competencies, networking and professional development opportunities are critical for early-career professionals. Many young HTA practitioners face difficulties in establishing connections within the field, accessing career advancement opportunities, and participating in international collaborations (11). Supporting early-career professionals in HTA is essential for ensuring growth and long-term sustainability of the field. By promoting and delivering mentorship, training, and career development initiatives, HTAi can help cultivate a new generation of HTA leaders who are well-equipped to navigate complex healthcare challenges. This can help early-career professionals build confidence in their analytical and decision-making skills while fostering HTA globally.

### Ethical issues in HTA

The Interest Group for Ethical Issues in HTA (EIG) was established initially by the International Network of Agencies in Health Technology Assessment, then converted into a formal HTAi IG in 2005. The EIG brings together those involved in HTA (including staff from HTA bodies, industry, government, academia, and patients and citizens) who have an interest in the explicit and formal identification and analysis of ethical issues pertaining to HTA. The EIG aims to establish and strengthen connections with others in related fields, for example, patient and citizen involvement in HTA and the role of philosophy in HTA; share good practices and related expertise in methodologies and tools for identifying and addressing ethical issues in HTA (12).

The dynamic nature of medical innovation requires ongoing attention to its potential ethical implications, addressing issues such as patient autonomy, equity, and the balance between individual and societal benefits, as highlighted by an article by the EIG leadership (13). The earliest contributions in the field emphasize that ethical inquiry in HTA is not optional, but a fundamental component, influencing how technologies are evaluated and implemented in healthcare systems. Values such as procedural and social justice, human rights, and public trust are intrinsically central to HTA evaluations and should be acknowledged and incorporated into HTA decision making accordingly. A structured approach to ethical evaluation enables healthcare systems to make more informed and justifiable decisions about certain technologies that may challenge societal norms. For instance, technologies used in neonatology may present unique ethical dilemmas, including decision making on life-sustaining treatments, resource allocation, and parental involvement (14). Similarly, ethical complexities may emerge in assessing advanced therapy medicinal products (ATMPs), such as gene and cell therapy, which often involve high costs, uncertain long-term outcomes, and difficult value judgments (15). A recent study highlighted how HTA bodies struggle with assessing these therapies due to their particular risk–benefit profiles and ethical implications (16). Another recent study published by the EIG leadership (17) highlighted a scenario where competencies for ethical analysis in HTA are often lacking or inconsistently applied. The authors emphasized that although ethical considerations are recognized as important within HTA, there are significant gaps in formal training, standardized methodologies, and the integration of ethical expertise into HTA processes. Their study outlined the core competencies necessary for ethics experts in HTA and defined essential skills, knowledge areas, and methodological approaches.

### Hospital-based HTA

Established in 2006, the Hospital-Based HTA Interest Group (HB-HTA IG) gathers professionals interested or involved in the use of an HTA logic at healthcare institutions for supporting both managerial and clinical decision-making processes. It provides a dialogue opportunity for members who are producers or users of HTA within hospitals; promotes awareness and knowledge of hospital-based HTA by sharing its principles, values, methods, and best practices; and identifies trends to support integration of national and hospital-based HTA programs. The collective experiences empower the role of hospital-based HTA in shaping the future of hospital healthcare systems globally.

Hospital decision makers, including managers and clinicians, are gatekeepers for many innovative technologies (18). Hospital-based HTA faces institutional, regulatory, and methodological challenges that require a specialized platform for discussion, collaboration, and knowledge exchange (19).

International experiences demonstrate that hospital-based HTA is evolving, with diverse models tailored to different healthcare systems. Institutionalization of hospital-based HTA often calls for cooperation among stakeholders, including policy makers, healthcare professionals, and industry representatives (20). The Adopting Hospital Based Health Technology Assessment (AdHopHTA) project was among the pioneers in the field, drawing on contributions from the HB-HTA-IG (21). It revealed that hospitals often struggle with resource constraints and the complexity of technology assessments, and that HTA in different hospital settings must be tailored subject to organizational structures, available financial and human resources, and other institutional challenges. Strategies to overcome these barriers have been proposed recently by the HB-HTA leadership, including the development of training programs, strengthening interdisciplinary collaboration, and integrating hospital-based HTA into national health policies (22).

### HTA in developing countries

Established in 2008, the HTA in Developing Countries IG (DCIG) aims to connect members interested in the early implementation of HTA in their countries. It aims to share experiences, activities, resources, and opportunities for education and training, and stimulate discussion about new ideas, needs, challenges, and solutions where HTA is in early development or adoption. Activities led by DCIG highlighted that patient communities may be key advocates for HTA uptake due to their interest in universal health coverage and fair and transparent methods for healthcare decision making.

Implementing HTA in low- or middle-income countries (LMICs) presents such challenges as limited institutional capacity, inadequate funding, and insufficient stakeholder engagement. Research led by DCIG leaders has highlighted that HTA adoption is limited by the absence of a well-defined policy framework and institutional support. In South Africa, for example, attempts to integrate HTA into the healthcare system have been hindered by fragmented decision making, a lack of standardized methodologies, and constraints in human resources (23). Similar issues were highlighted in parts of Eastern Europe and Central Asia where political instability, capacity shortages, and fragmented data sources posed systemic barriers to the successful implementation of HTA (24). Although not specific to LMICs, achieving stakeholder engagement can be especially challenging in those contexts. The work of DCIG has shown that patient/public involvement remains limited in LMICs due to a lack of awareness, inadequate communication channels, and cultural barriers (25). International collaboration, including technical support, is essential to overcome these barriers and scale up HTA capacity in LMICs (26).

### Information retrieval

Since its inception in 2005, the Information Retrieval Interest Group (IRG) has been convening members with an active interest in information resources for HTA, sharing expertise, practical experience, and related tools and processes for advancing literature searching and synthesis.

HTA relies on high-quality evidence synthesis to inform policy and clinical decision making. At its core is information retrieval, a critical yet often underappreciated discipline that ensures comprehensive, accurate, and unbiased identification of relevant literature. The increasing complexity of health information sources and evolving methodologies in evidence synthesis highlight the value of information specialists in planning, conducting, and reporting systematic reviews in HTA. However, although statisticians and other professionals are routinely involved and acknowledged in systematic reviews, information specialists are often underutilized (27). International initiatives have demonstrated the value of shared expertise and harmonized approaches in conducting literature searches. The work done within the European Network for Health Technology Assessment in the development of collaborative methods, processes, and infrastructures to improve information retrieval across European HTA agencies deserves to be mentioned (28). Peer review and quality assurance in HTA information retrieval and synthesis have also improved transparency, reproducibility, and validity in the field (29). IRG initiatives such as the SuRe Info (Summarized Research in Information Retrieval for HTA) framework (30) provide updated guidance on evidence-based searching for HTA professionals.

### Medical devices

The Medical Devices Interest Group (MDIG), established in 2019, aims to ensure that best practices in HTA of medical nondrug technologies are shared, providing a space for all stakeholders to discuss the strengths, weaknesses, challenges, and opportunities of introducing medical device innovation into healthcare systems under the responsibility of increasing patient outcomes and lowering system costs.

Medical devices encompass a broad spectrum, ranging from simple instruments to complex digital systems, each with distinct developmental, regulatory, and clinical considerations. Assessment of medical devices is distinguished by devices’ iterative development and shorter life cycles, which often result in limited availability of long-term clinical data at the time of evaluation. Traditional HTA frameworks, primarily designed for pharmaceuticals, typically do not adequately capture the dynamic nature of medical device innovation. This underscores the need for adapted assessment methodologies that can accommodate continuous improvements and real-world performance data (31). In fact, a recent review (32) indicated that HTA methods, guidelines, and reports often lack comprehensive inclusion of medical device-specific characteristics. This omission can lead to assessments that do not fully account for factors such as user dependency, learning curves, and context-specific performance, potentially diminishing the accuracy and relevance of HTA findings for medical devices. Moreover, organizational and contextual differences in device deployment can confound device assessment (33). Furthermore, national and international regulation of medical devices is evolving, with increasing emphasis on postmarket evidence generation and continuous monitoring to increase safety and effectiveness, especially for high-risk devices (34). Additionally, the increasing integration of digital health solutions further complicates medical device assessment. Challenges in evaluating digital medical devices highlight (35) the need for specialized HTA approaches to assess these technologies, including such factors as cybersecurity, real-world data integration, and patient engagement (35).

### Patient and citizen involvement

The Patient and Citizen Involvement in HTA Interest Group (PCIG) was established in 2005 to promote and develop robust methodologies to incorporate these perspectives into HTAs and share best practices in participation in HTA. This includes co-developing recommendations for good practice (36) and promoting consistent terminology to aid understanding and research. Since 2010, the IG has defined patient involvement as two complementary but equally important approaches: (i) research into patient aspects: needs, preferences, experiences, and perspectives and (ii) patient participation in HTA, across individual HTAs, overarching processes, and HTA policy (37). As involvement varies across jurisdictions due to the essential influence of goals and settings, PCIG has led the development of practical tools to support uptake, such as Delphi-study-derived *HTAi Values and Quality Standards for Patient Involvement* and submission and summary templates that have been translated and adapted for local needs internationally (38).

It has been argued that framing HTA through patient perspectives leads to a more holistic understanding of what constitutes value in healthcare as, beyond clinical effectiveness and cost-effectiveness, patients often emphasize treatment accessibility, disease burden, and the psychosocial impact of interventions (39). Although studies have highlighted the contribution of patient knowledge to HTA (40), especially in addressing gaps and uncertainties in the traditional evidence or validating evidence and assumptions, its impact can be unclear. Beyond the challenges of transparency and the multiple domains considered in HTA deliberations, the PCIG’s research highlighted that a lack of evaluation (41) and different stakeholder perspectives of impact (42) are barriers to understanding patient involvement’s influence. In response, the PCIG undertook a study to collate stories of impact from the perspectives of patients, technology developers, and HTA bodies. An analysis of these stories identified three domains of impact (on the patient/patient organization, on the HTA body, and on the HTA recommendations or results), which form a practical framework to support increased evaluation in the field (43).

Despite the potential benefits of patient involvement in HTA, its uptake varies. Patient participation is more widely employed than undertaking research into patient aspects or integrating published evidence in this area. Another PCIG study suggested that HTA bodies were unsure of how to make use of patient preference data (44). An enduring question is whether HTA processes are inherently limited in incorporating patient knowledge because patient perspectives have usually been absent during process and method development. A PCIG study of patient involvement at the organizational level of HTA in five jurisdictions observed growing patient involvement in instances where patients had participated in governance, defining patient involvement processes, developing HTA processes and methods, and/or capacity building (45).

### Public health

The Public Health IG (PHIG) is dedicated to raising awareness of the importance of HTA in (i) evaluating public health interventions for promoting health, preventing diseases and preserving and/or ensuring sustainable healthcare and social infrastructures; (ii) facilitating the development and application of appropriate methodologies and HTA approaches in the area of public health; and (iii) engaging with diverse stakeholders to strengthen intrasectorial cooperation of major importance in the area of HTA and public health. The PHIG was established in 2019 by consolidating the Initiatives for Public Health Outcomes Research and Measurement IG and the Assessment of Vaccines Programs IG.

HTA has traditionally focused on pharmaceuticals and medical devices, often neglecting the evaluation of broader public health measures such as vaccination programs, infection control strategies, and digital health interventions, partly due to difficulties in quantifying benefits such as disease prevention, health promotion, and economic savings from reduced healthcare utilization (46). Certainly, the COVID-19 pandemic has exemplified the importance of integrating HTA into public health decision making (47). Vaccination programs are among the most impactful public health interventions, yet their assessment presents particular challenges, as factors such as herd immunity, epidemiological trends, and long-term cost savings must be considered (48). Another urgent issue in HTA of public health concerns the growing threat of antimicrobial resistance (AMR), including discrepancies in how new antibiotics are assessed and valued across different national HTA agencies.

Given the global nature of AMR, there is a need for coordinated HTA approaches that incentivize antibiotic development while promoting responsible stewardship (49). The rise of digital public health interventions, such as mobile health apps, telemedicine platforms, and digital disease surveillance tools, further necessitates specialized HTA approaches where usability, scalability, interoperability, and data security need to be considered (50).

### Rare diseases

The Rare Diseases IG (RDIG), the newest HTAi IGs, was established in 2023 to address the challenges that arise in determining value in technologies for diseases that are often genetic, of predominantly childhood onset, and chronic. The RDIG shares good practices in the generation and interpretation of evidence in rare disease populations and explores how HTA can consider wider aspects of value in rare disease populations within the context of fairness to society. The RDIG aims to develop awareness of the specific challenges associated with studying rare disease populations, not just in terms of small populations but in understanding natural history and disease progression, and paucity of knowledge about optimal endpoints given complex disease manifestations. The initial focus of RDIG is on four main areas: (i) dissemination of relevant information and initiatives in HTA of rare diseases, (ii) characterization of the issues with rare diseases and their implications for HTA, (iii) understanding evidence generation and interpretation in the context of the rare diseases complexity, and (iv) economic modeling methods used to assess treatments for rare diseases.

Given the increasing availability of high-cost treatments for rare conditions, ensuring that HTA frameworks account for the specificities of rare disease evaluation is essential. A recent German study (51) highlighted that orphan drugs frequently fail to show added benefit under the local HTA process due to evidence gaps, leading to persistent unmet medical needs. This underscores the need for innovative assessment approaches, such as the use of RWE, adaptive clinical trials, and surrogate endpoints, to ensure that promising treatments are not dismissed due to methodological limitations. Relevant inconsistencies in reimbursement policies have been reported across different healthcare systems in Europe (52) and other contexts (53), where some systems prioritize cost-effectiveness, whereas others emphasize broader social and ethical considerations. All of these disparities in clinical and nonclinical evaluation criteria affect access to treatment. Value assessment frameworks tailored to rare diseases have been proposed, incorporating such factors as disease severity, treatment innovation, and societal impact to better capture the value of orphan drugs (54). Additionally, there is growing recognition that societal preferences, including ethical and equity considerations, must be integrated into rare disease HTA (55).

### Real-world evidence and artificial intelligence

The Real-world Evidence and Artificial Intelligence Interest Group (RWE&AI IG) was established in 2020 to serve as a discussion forum where members can provide insights, exchange ideas, and make collective decisions about subjects related to the RWE and artificial intelligence (AI). The RWE&AI IG aims to: (i) educate evidence developers and assessors, including practitioners, researchers and regulators, on how AI and machine learning (ML) are and will be used, and on RWE generation and utilization in the context of regulatory and HTA dossiers; (ii) identify and engage people with interest and expertise in RWE, AI, and HTA; and (iii) explore further use in collaborative projects, including facilitation of cross-border and interdisciplinary collaboration and identify early cases of best practice.

The integration of RWE into AI-driven methodologies presents both opportunities and challenges for HTA processes. RWE offers insights into the long-term effectiveness, safety, and cost-effectiveness of health technologies in routine clinical practice. However, its use is still met with methodological and regulatory challenges related to collection, analysis, and quality standards (56), and concerns about data quality, standardization, and transparency have been highlighted as typical barriers to incorporating RWE in HTA (57). One of the key issues in leveraging RWE is harmonizing data from different sources, such as electronic health records, registries, and claims databases, to improve the robustness of HTA. In this context, inconsistencies in data collection and reporting remain significant obstacles (58). In parallel, technologies that are enabled by AI-driven predictive modeling, risk stratification, and personalization of medicine are changing how evidence is generated, which then in turn is transforming how evidence is evaluated in HTA. However, algorithmic bias, transparency, and regulatory approval raise concerns requiring improved validation methodologies and clearer guidelines (59). In the meantime, as AI is already integral to many healthcare technologies, existing HTA frameworks may be unsuitable for assessing these products. Validated frameworks for assessing AI-enabled technologies may prove to be critical for ensuring their safe and effective implementation. Furthermore, these technologies would benefit from dynamic HTA approaches that can account for continuous algorithm updates and related changes (60).

## Shared domains of actions and cross-cutting topics

Given their respective remits and memberships, the IGs have common interests. Some IGs emphasize the importance of education, mentorship, and professional growth. The Early Career Network plays a pivotal role in developing future HTA professionals, whereas the Hospital-Based HTA IG and the HTA in Developing Countries IG focus on enhancing institutional capacities respectively in the hospital setting and in countries where HTA is only emerging. Similarly, the Information Retrieval IG convenes a specific group of professionals, those involved in evidence searching. Some IGs have tailored their scope around stakeholder engagement and inclusivity, such as the Patient and Citizen Involvement IG, who champion the integration of patient perspectives in HTA decision making, a theme that resonates with the ethical considerations explored by the Ethical Issues in HTA IG and aligns with the aim of Public Health IG to advocate for a more holistic evaluation of health technologies that includes societal and equity considerations. Innovation, methodological advancements, and adaptation of HTA are emphasized among the RWE&AI, Medical Devices, and Rare Diseases IGs. The interconnected nature of IGs’ themes with the HTA ecosystem positions them to drive collective impact in advancing HTA methodologies and applications globally. For example, AI applications in HTA are a rapidly growing area, where multiple IGs can explore its role in evidence generation, predictive analytics, and automation in decision-making. Lifecycle approaches in HTA are being promoted in a context of rapidly changing scenarios of continuous evidence collection and reassessment to keep pace with technological change. Environmental impact in HTA is another emerging concern across several IGs as the environmental footprint of health technologies, including sustainability considerations in medical devices and pharmaceuticals, could become a key area for cross-IG collaboration.

The set of shared themes can expand with, for example:

- equity and access to health technologies, to ensure that HTA frameworks incorporate principles to serve specific, underserved population groups;
- digital health and telemedicine, where the rise of telehealth and digital therapeutics is prompting regulatory adaptation and calls for assessment of their long-term effectiveness and accessibility; and
- patient-centered healthcare, where current HTA frameworks are under revision to consider meaningful inclusion of patient-centered outcomes and broader value propositions beyond cost-effectiveness.

## Support, challenges, and critical reflections

HTAi has established formal governance mechanisms to guide the formation, operation, and dissolution of IGs. Oversight is provided by the HTAi Secretariat and the Board of Directors, with IGs created through a structured process based on member-driven proposals aligned with the society’s strategic priorities. IGs may be restructured or dissolved if they become inactive or if their focus no longer reflects evolving needs. All IG activities are governed by Board-approved policy, and each IG is required to maintain a terms of reference outlining roles, responsibilities, and procedures for leadership appointments and elections – also subject to Board approval. Recognizing the importance of administrative continuity, HTAi assigns a technical officer to each IG. Typically, a student or early-career professional, the technical officer is contracted by HTAi to provide support while benefiting from professional development opportunities as part of the role.

To support IG-led initiatives, HTAi conducts two funding calls per year. Through these competitive processes, IGs may submit project proposals and receive partial or full funding based on merit and strategic alignment. Since this year, an annual report presenting the projects, webinars, and other initiatives and activities of the HTAi IGs is available for download and consultation from the HTAi web site (61).

In parallel, HTAi also establishes Working Groups, distinct from IGs, with clearly defined, time-limited mandates to produce specific outputs or complete targeted projects. This dual structure enables HTAi to balance long-term, community-driven development through IGs with focused, agile responses to emerging needs via working groups.

Although IGs have become vital platforms for the society, their continued success requires grappling with several persistent challenges. A primary concern is internal engagement. Although IGs often boast large and diverse memberships (often numbering in the hundreds, exceeding 500 in some cases), active participation is typically concentrated among a small core of volunteers. Many members join primarily to access resources or stay informed, but do not engage as co-creators or collaborators in shaping outputs, leading to an imbalance between consumption and contribution. This limited participation narrows the diversity of voices in discussions and can place a disproportionate burden on a few highly active members, potentially reducing the groups’ responsiveness to emerging issues. Resource constraints are a recurring challenge. Although IGs may apply for HTAi funds through a competitive process, access to consistent and scalable funding remains limited. Structured guidance and processes to attract external funding are under development but are not yet fully implemented. A few IGs with experienced leadership and strategic positioning have been successful in securing external resources, but many others operate with minimal financial support. The ability to mobilize funding remains a key differentiator in an IG’s capacity to plan and execute impactful work.

Efficiency is another pressing concern. All IG contributors are volunteers, and their engagement must compete with professional and personal commitments. This often results in delays in delivering outputs, fulfilling administrative requirements, or maintaining momentum on long-term initiatives. Recognizing this constraint, recent policy changes have enabled IGs to externalize selected tasks to contractors using approved funds. This shift offers a new pathway to enhance productivity and meet project timelines while preserving the volunteer-driven ethos of the groups. A further challenge has been limited information exchange between IGs. Historically, IGs have operated in relative isolation, often unaware of parallel work or opportunities for synergy. This siloed structure has constrained cross-group learning and reduced the potential for collaborative innovation. In response, the HTAi Secretariat is actively working to improve coordination by facilitating mutual exchanges, supporting shared initiatives, and creating opportunities for cross-IG engagement through joint sessions, communications, and strategic planning. These include regular sharing of project scoping and outcomes at the IG Steering Committee, comprised IG co-chairs.

Compared to thematic groups in other societies, HTAi’s IGs are notable for their inclusive, multistakeholder composition and member-initiated focus areas. Although this bottom-up structure encourages relevance and responsiveness to the field, it also brings challenges in ensuring consistency, sustained productivity, and global representation.

## Conclusions

The HTAi IGs are well positioned to serve as pillars shaping the future of HTA by fostering international collaboration, driving methodological advancements, and enhancing stakeholder inclusion. These are implemented by active, interdisciplinary, global engagement of diverse members. Problem-solving and shared learning are core to the remits of the IGs and essential to the mission of HTAi. HTA is highly dependent on egalitarian stakeholder engagement, and HTAi provides a collegial space for professionals at all career stages who seek to collaborate in advancing the field. The IGs will continue to produce high-quality publications, policy recommendations, and conference sessions for widespread dissemination of best practices. The IGs have demonstrated their value as vibrant communities of practice but maximizing their potential will require a renewed focus on equitable participation, cross-group collaboration, sustainable resourcing, and structured support. Strategic efforts by HTAi leadership, Secretariat coordination, and shared responsibility among members will be critical to advancing the next phase of IG development and ensuring their continued relevance and effectiveness within the global HTA landscape.

## References

[1] O’Rourke B, Oortwijn W, Schuller T. The new definition of health technology assessment: A milestone in international collaboration. Int J Technol Assess Health Care. 2020;36(3):187–190. 32398176 10.1017/S0266462320000215

[2] Banta D, Jonsson E, Childs P. History of the international societies in health technology assessment: International Society for Technology Assessment in health care and health technology assessment international. Int J Technol Assess Health Care. 2009;25 Suppl 1:19–23. doi: 10.1017/S0266462309090369. 19505349

[3] Trowman R, Migliore A, Ollendorf DA. Health technology assessment 2025 and beyond: Lifecycle approaches to promote engagement and efficiency in health technology assessment. Int J Technol Assess Health Care. 2023;39(1):e15. doi: 10.1017/S0266462323000090. 36815310 PMC11574536

[4] Pichler FB, Boysen M, Mittmann N, et al. Lifecycle HTA: Promising applications and a framework for implementation. *An HTAi global policy forum task force report*. Int J Technol Assess Health Care. 2024;40(1):e50. doi: 10.1017/S0266462324000187. 38727087 PMC11569898

[5] Gutiérrez-Ibarluzea I, Chiumente M, Dauben HP. The life cycle of health technologies. Challenges and ways forward. Front Pharmacol. 2017 24;8:14. doi: 10.3389/fphar.2017.00014. PMC5258694 28174538

[6] Calabrò GE, La Torre G, de Waure C, et al. Disinvestment in healthcare: An overview of HTA agencies and organizations activities at European level. BMC Health Serv Res. 2018;18(1):148. 29490647 10.1186/s12913-018-2941-0 PMC5831213

[7] Cadeddu C, Regazzi L, Di Brino E, et al. The added value of applying a disinvestment approach to the process of health technology assessment in Italy. Int J Technol Assess Health Care. 2023;39(1):e17. doi: 10.1017/S0266462323000107. 36861658 PMC11570134

[8] Esandi ME, Gutiérrez-Ibarluzea I, Ibargoyen-Roteta N, Godman B. An evidence-based framework for identifying technologies of no or low-added value (NLVT). Int J Technol Assess Health Care. 2020;36(1):50–57. doi: 10.1017/S0266462319000734. 31831086

[9] HTAi website. The newcomer’s guide to HTA. Available from: https://htai.org/learn-with-us/newcomers-guide/.

[10] Mueller D, Gutierrez-Ibarluzea I, Chiumente M, Oortwijn W. Toward a common understanding of competencies for health technology assessment: Enhancing educational and training programs around the globe. Int J Technol Assess Health Care. 2020;37:e29 doi: 10.1017/S0266462320001919. 33280625

[11] Migliore A, Mueller D, Oortwijn W. The newcomer’s guide to health technology assessment: A collection of resources for early career professionals. Int J Technol Assess Health Care. 2024;40(1):e38. doi: 10.1017/S0266462324000424. 39494828 PMC11563173

[12] Single AN, Scott AM, Wale J. Developing guidance on ethics for patient groups collecting and reporting patient information for health technology assessments. Patient. 2016;9(1):1–4. doi: 10.1007/s40271-015-0143-y. 26476960

[13] Refolo P, Duthie K, Hofmann B, et al. Ethical challenges for health technology assessment (HTA) in the evolving evidence landscape. Int J Technol Assess Health Care. 2024;40(1):e39. doi: 10.1017/S0266462324000394. 39494823 PMC11569911

[14] Di Pietro ML. Application of health technology assessment: A new need for an ethical neonatology. Eur J Pediatr. 2024;183(10):4179–4184. doi: 10.1007/s00431-024-05620-5. 39136757

[15] Gonçalves E. Advanced therapy medicinal products: Value judgement and ethical evaluation in health technology assessment. Eur J Health Econ. 2020;21(3):311–320. doi: 10.1007/s10198-019-01147-x. 31919703 PMC7188714

[16] Drummond M, Ciani O, Fornaro G, et al. How are health technology assessment bodies responding to the assessment challenges posed by cell and gene therapy? BMC Health Serv Res. 2023;23(1):484. doi: 10.1186/s12913-023-09494-5. 37179322 PMC10182681

[17] Refolo P, Bond K, Bloemen B, et al. Core competencies for ethics experts in health technology assessment. Int J Technol Assess Health Care. 2020;36(6):534–539. doi: 10.1017/S0266462320001968. 33292881

[18] Martin T, Guercio A, Besseau H, et al. Hospital-based health technology assessment of innovative medical devices: Insights from a nationwide survey in France. Int J Technol Assess Health Care. 2023;39(1):e58. doi: 10.1017/S0266462323000521. 37732461 PMC11570185

[19] Galdino JPDS, Camargo EB, Elias FTS. Sedimentation of health technology assessment in hospitals: A scoping review. Cad Saude Publica. 2021;37(9):e00352520: 10.1590/0102-311X00352520. 34586173

[20] Gałązka-Sobotka M, Kowalska-Bobko I, Lach K, Mela A, Furman M, Lipska I. Recommendations for the implementation of hospital based HTA in Poland: Lessons learned from international experience. Front Pharmacol. 2021;11:594644. 10.3389/fphar.2020.594644. 34054508 PMC8155722

[21] Cicchetti A, Iacopino V, Coretti S, et al. Toward a contingency model for hospital-based health technology assessment: Evidence from ADHOPHTA project. Int J Technol Assess Health Care. 2018;34(2):205–211. doi: 10.1017/S0266462318000119. 29656722

[22] Lipska I, Di Bidino R, Niewada M, et al. Overcoming barriers in hospital-based health technology assessment (HB-HTA): International expert panel consensus. Healthcare (Basel). 2024;12(9):889. doi: 10.3390/healthcare12090889. 38727447 PMC11083158

[23] Mueller D. Addressing the challenges of implementing a health technology assessment policy framework in South Africa. Int J Technol Assess Health Care. 2020;13:1–6. doi: 10.1017/S0266462320000562. 32787995

[24] Migliore A, Vicari N, Turk E, Sucu R. Driving policy dialogue on health technology assessment in Eastern Europe and Central Asia: Reporting from an initiative of health technology assessment international. Int J Technol Assess Health Care. 2025 6:1–20. doi: 10.1017/S0266462325000066. PMC11894386 39910984

[25] Holtorf AP, Mueller D, Sousa MSA, et al. Pilot approach to analyzing patient and citizen involvement in health technology assessment in four diverse low- and middle-income countries. Int J Technol Assess Health Care. 2021;37:e1. doi: 10.1017/S0266462320002263. 33491616

[26] Mueller D, Alouane L, Jameleddine M, Lenoir-Wijnkoop I. Scaling up health technology assessment capacities in selected African countries – a conceivable route ahead. Int J Technol Assess Health Care. 2023;39(1):e9. doi: 10.1017/S0266462323000016. 36710506 PMC11574544

[27] Waffenschmidt S, Bender R. Involvement of information specialists and statisticians in systematic reviews. Int J Technol Assess Health Care. 2023;39(1):e22. 10.1017/S026646232300020X. 37096439 PMC11570075

[28] Waffenschmidt S, van Amsterdam-Lunze M, Gomez RI, Rehrmann M, Harboe I, Hausner E. Information specialist collaboration in Europe: Collaborative methods, processes, and infrastructure through EUnetHTA. Int J Technol Assess Health Care. 2020;37:e20. doi: 10.1017/S0266462320000732. 33081862

[29] Lefebvre C, Duffy S. Peer review of searches for studies for health technology assessments, systematic reviews, and other evidence syntheses. Int J Technol Assess Health Care. 2021;37(1):e64. doi: 10.1017/S0266462321000210. 34024305

[30] Isojärvi J, Glanville J. Evidence-based searching for health technology assessment: Keeping up to date with SuRe info. Int J Technol Assess Health Care. 2021;37(1):e51. doi: 10.1017/S026646232100009X. 33840393

[31] Schnell-Inderst P, Mayer J, Lauterberg J, et al. Health technology assessment of medical devices: What is different? An overview of three European projects. Z Evid Fortbild Qual Gesundhwes. 2015;109(4–5):309–18. 10.1016/j.zefq.2015.06.011. 26354131

[32] Basu R, Eggington S, Hallas N, Strachan L. Are medical device characteristics included in HTA methods guidelines and reports? A brief review. Appl Health Econ Health Policy. 2024;22(5):653–664. doi: 10.1007/s40258-024-00896-y. 38965161

[33] Bloemen B, Oortwijn W. Assessing medical devices: A qualitative study from the validate perspective. Int J Technol Assess Health Care. 2024;40(1):e29. doi: 10.1017/S0266462324000254. 38654522 PMC11569912

[34] Aranda J, Dobrzynska A, Rosario-Lozano MP, Rejón-Parrilla JC, Epstein D, Blasco-Amaro JA. Regulatory perspectives on post-market evidence generation schemes for high-risk medical devices: A systematic review. Expert Rev Pharmacoecon Outcomes Res. 2024;1:1–15. doi: 10.1080/14737167.2024.2431234. 39618103

[35] Trancart A, Riche VP, Disset A, et al. Evaluation of digital medical devices: How to take into account the specificities of these solutions? Therapie. 2024;79(1):137–150. doi: 10.1016/j.therap.2024.01.001. 38307754

[36] Holtorf AP, Bertelsen N, Jarke H, Dutarte M, Scalabrini S, Strammiello V. Stakeholder perspectives on the current status and potential barriers of patient involvement in health technology assessment (HTA) across Europe. Int J Technol Assess Health Care. 2024;40(1):e81. doi: 10.1017/S0266462324004707. 39698996 PMC11703633

[37] Facey K, Boivin A, Gracia J, et al. Patients’ perspectives in health technology assessment: A route to robust evidence and fair deliberation. Int J Technol Assess Health Care. 2010;26(3):334–40. doi: 10.1017/S0266462310000395. 20584364

[38] HTAi website. HTAi interest sub-Group for Patient/citizen involvement in HTA. Values and quality standards for patient involvement in HTA. 2014. Available from: https://past.htai.org/wp-content/uploads/2018/02/PCISG-Info-ValuesandStandards-30-Jun14.pdf?_gl=1*1dhgenv*_ga*MTM2MjA5MjAxOS4xNzEwNTMyMDU4*_ga_79CPBECN0V*MTcxMTIwMjcwMy43LjEuMTcxMTIwMjczNC4wLjAuMA.

[39] Gunn CJ, Regeer BJ, Zuiderent-Jerak T. A HTA of what? Reframing through including patient perspectives in health technology assessment processes. Int J Technol Assess Health Care. 2023;39(1):e27. doi: 10.1017/S0266462323000132. 37198925 PMC11570000

[40] Livingstone H, Verdiel V, Crosbie H, et al. Evaluation of the impact of patient input in health technology assessments at NICE. Int J Technol Assess Health Care. 2023;37(1):e33. 10.1017/S0266462320002214 33509314

[41] Weeks L, Polisena J, Scott AM, et al. Evaluation of patient and public involvement initiatives in health technology assessment: A survey of international agencies. Int J Technol Assess Health Care. 2017;33(6):715–723. 29122048 10.1017/S0266462317000976

[42] Single ANV, Facey KM, Livingstone H, Silva AS. Stories of patient involvement impact in health technology assessments: A discussion paper. Int J Technol Assess Health Care. 2019;35(4):266–272. doi: 10.1017/S0266462319000552. 31337453

[43] Lopez Gousset V, Silveira Silva A, Holtorf AP, Toledo-Chávarri A, Single A. The three-domain impact framework for characterizing impact of patient involvement in health technology assessment. Int J Technol Assess Health Care. 2024;40(1):e52. doi: 10.1017/S0266462324000400. 39523886 PMC11579675

[44] Germeni E, Fifer S, Hiligsmann M, et al. A genuine need or nice to have? Understanding HTA representatives’ perspectives on the use of patient preference data. Int J Technol Assess Health Care. 2024;40(1):e60. doi: 10.1017/S026646232400463X. 39562326 PMC11579672

[45] Nabarette H, Chastenay MH, Dupont JK, Ganache I, Single ANV. Patient and citizen participation at the organizational level in health technology assessment: An exploratory study in five jurisdictions. Int J Technol Assess Health Care. 2023;39(1):e51. doi: 10.1017/S0266462323000417. 37551103 PMC11570056

[46] Stojanovic J, Wübbeler M, Geis S, Reviriego E, Gutiérrez-Ibarluzea I, Lenoir-Wijnkoop I. Evaluating public health interventions: A neglected area in health technology assessment. Front Public Health. 2020;8:106. doi: 10.3389/fpubh.2020.00106. 32391300 PMC7188782

[47] Hasdeu S, Beliera A, Alvarez J, Sánchez Viamonte J. Exploring the linkage between health technology assessment and decision making during COVID-19 public health emergency in a developing country: Analysis of processes and results. Int J Technol Assess Health Care. 2024;40(1):e42. doi: 10.1017/S0266462324000473. 39494834 PMC11563179

[48] Largeron N, D’Agostino P, Chapman R, et al. Guiding principles for evaluating vaccines in joint health technology assessment in the European Union: Preparing for the European Union’s regulation on health technology assessment for vaccines. Value Health. 2024;27(10):1318–1327. doi: 10.1016/j.jval.2024.06.011. 38977187

[49] Dumont R, Lengliné E, Delorme C, et al. How do we respond to the threat of multidrug-resistant bacteria? Comparison of antibiotic appraisals from 2016 to 2020 of the French, English, and German HTA agencies. Int J Technol Assess Health Care. 2024;40(1):e72. doi: 10.1017/S0266462324000552. 39651580 PMC11703620

[50] Gudi N, Raj EA, Jahn B, Siebert U, Brand A. Evaluations of digital public health interventions in the WHO Southeast Asia region: A systematic literature review. Int J Technol Assess Health Care. 2024;40(1):e78. doi: 10.1017/S026646232400045X. 39690747 PMC11703627

[51] Kranz P, McGauran N, Ünal C, Kaiser T. Results of health technology assessments of orphan drugs in Germany-lack of added benefit, evidence gaps, and persisting unmet medical needs. Int J Technol Assess Health Care. 2024;40(1):e68. doi: 10.1017/S026646232400062X. 39623907 PMC11703625

[52] Jakubowski S, Holko P, Nowak R, et al. Clinical and non-clinical aspects of reimbursement policy for orphan drugs in selected European countries. Front Pharmacol. 2024;15:1498386. doi: 10.3389/fphar.2024.1498386. 39629081 PMC11611580

[53] Felippini A, Biglia LV, Lima TM, Aguiar PM. HTA criteria adopted in different models of public healthcare systems for orphan drugs: A scoping review. Health Policy. 2024;144:105080. doi: 10.1016/j.healthpol.2024.105080. 38733643

[54] Biglia LV, Felippini A, Ribeiro TB, Lima TM, Aguiar PM. Development of a value assessment framework for health technology assessment in rare diseases drugs: Insights from a Delphi study in Brazil. Int J Technol Assess Health Care. 2025;41(1):e6. doi: 10.1017/S0266462324004835. 39783027 PMC11811954

[55] Vásquez P, Hall L, Merlo G. Societal preferences in health technology assessments for rare diseases and orphan drugs: A systematic literature review of new analytic approaches. Value Health Reg Issues. 2024;44:101026. doi: 10.1016/j.vhri.2024.101026. 39059264

[56] Oortwijn W, Sampietro-Colom L, Trowman R. How to Deal with the inevitable: Generating real-world data and using real-world evidence for HTA purposes – from theory to action. Int J Technol Assess Health Care. 2019;35(4):346–350. doi: 10.1017/S0266462319000400. 31198129

[57] Zisis K, Pavi E, Geitona M, Athanasakis K. Real-world data: A comprehensive literature review on the barriers, challenges, and opportunities associated with their inclusion in the health technology assessment process. J Pharm Pharm Sci. 2024;27:12302. doi: 10.3389/jpps.2024.12302. 38481726 PMC10932954

[58] Graili P, Guertin JR, Chan KKW, Tadrous M. Integration of real-world evidence from different data sources in health technology assessment. J Pharm Pharm Sci. 2023;26:11460. doi: 10.3389/jpps.2023.11460. 37529633 PMC10387532

[59] Zemplényi A, Tachkov K, Balkanyi L, et al. Recommendations to overcome barriers to the use of artificial intelligence-driven evidence in health technology assessment. Front Public Health. 2023;11:1088121. doi: 10.3389/fpubh.2023.1088121. 37181704 PMC10171457

[60] Di Bidino R, Daugbjerg S, Papavero SC, Haraldsen IH, Cicchetti A, Sacchini D. Health technology assessment framework for artificial intelligence-based technologies. Int J Technol Assess Health Care. 2024;40(1):e61. doi: 10.1017/S0266462324000308. 39568412 PMC11703629

[61] Health Technology Assessment international (HTAi). Interest groups section. Available from: https://htai.org/engage-with-us/interest-groups/.

